# Natural approach of using nisin and its nanoform as food bio-preservatives against methicillin resistant *Staphylococcus aureus* and *E.coli* O157:H7 in yoghurt

**DOI:** 10.1186/s12917-024-03985-1

**Published:** 2024-05-11

**Authors:** Walaa M. Elsherif, Alshimaa A. Hassanien, Gamal M. Zayed, Sahar M. Kamal

**Affiliations:** 1 Certified Food Lab, Nanotechnology Research and Synthesis Unit, Animal Health Research Institute (AHRI), Agriculture Research Center (ARC), Assiut,, Egypt; 2 Faculty of Health Sciences Technology, New Assiut Technological University (NATU), Assiut, Egypt; 3 Department of Zoonoses, Faculty of Veterinary Medicine, Sohag University, Sohag, Egypt; 4 Department of Pharmaceutics and Pharmaceutical Technology, Al-Azhar University, Assiut, Egypt; 5 Department of Food Hygiene, Safety and Technology, Faculty of Veterinary Medicine, Assiut University, Assiut, Egypt

**Keywords:** Yoghurt, MRSA, *E.coli* O157:H7, Nisin, Nanoparticles, Cytotoxicity, Food preservative

## Abstract

**Background:**

Natural antimicrobial agents such as nisin were used to control the growth of foodborne pathogens in dairy products. The current study aimed to examine the inhibitory effect of pure nisin and nisin nanoparticles (nisin NPs) against methicillin resistant *Staphylococcus aureus* (MRSA) and *E.coli* O157:H7 during the manufacturing and storage of yoghurt. Nisin NPs were prepared using new, natural, and safe nano-precipitation method by acetic acid. The prepared NPs were characterized using zeta-sizer and transmission electron microscopy (TEM). In addition, the cytotoxicity of nisin NPs on vero cells was assessed using the 3-(4,5-Dimethylthiazol-2-yl)-2,5-diphenyltetrazolium bromide (MTT) assay. The minimum inhibitory concentrations (MICs) of nisin and its nanoparticles were determined using agar well-diffusion method. Further, fresh buffalo’s milk was inoculated with MRSA or *E.coli* O157:H7 (1 × 10^6^ CFU/ml) with the addition of either nisin or nisin NPs, and then the inoculated milk was used for yoghurt making. The organoleptic properties, pH and bacterial load of the obtained yoghurt were evaluated during storage in comparison to control group.

**Results:**

The obtained results showed a strong antibacterial activity of nisin NPs (0.125 mg/mL) against MRSA and *E.coli* O157:H7 in comparison with control and pure nisin groups. Notably, complete eradication of MRSA and *E.coli* O157:H7 was observed in yoghurt formulated with nisin NPs after 24 h and 5th day of storage, respectively. The shelf life of yoghurt inoculated with nisin nanoparticles was extended than those manufactured without addition of such nanoparticles.

**Conclusions:**

Overall, the present study indicated that the addition of nisin NPs during processing of yoghurt could be a useful tool for food preservation against MRSA and *E.coli* O157:H7 in dairy industry.

## Introduction

Using of bacteriocins such as nisin alone or combined with other natural materials such as essential oils, could be represented as a useful candidate for improving the microbiological quality and maintaining the sensory properties of milk and milk products [1, 2]. The utility of nisin as a bio preservative in food industry has been approved and this bacteriocins was effective enough to extended shelf life in regions with inadequate preservation facilities such as developing countries [3]. Nisin is a natural water-soluble antibacterial peptide (AMP) composed of 34 amino acid residues produced by *Lactococcus lactis.* It has the ability to inhibit the growth of some foodborne pathogens and many of Gram-positive spoilage bacteria [4, 5]. This antibacterial peptide is generally regarded as a safe food preservative by the joint Food and Agriculture Organization and World Health Organization (FAO/WHO), also by the US Food and Drug Administration (FDA) [6, 7]. Based on aforementioned permissions, it is widely commercialized as a safe and natural food preservative in the food industry in more than 50 countries around the world [8].

The antibacterial activity of nisin in food is depending on several factors such as its solubility, pH and structural properties of target bacteria. It could exhibit potent antimicrobial activities against many species of Gram-positive pathogens, while it has little effect against Gram-negative bacteria, yeast and fungi due to their outer membrane barriers [9]. The exact antibacterial mechanism of nisin is attributed to the passage of nisin through the cell wall of bacteria and its interaction with lipid II, which considered as an essential element in the bacterial cell wall [9].

There are some obstacles that can hinder the antimicrobial efficacy of free nisin as a food bio preservative such as its ability to interact with food components (e.g. proteolytic enzymes, phospholipids, fatty acids and proteins), high pH and many other food additives. These factors could drastically reduce or completely diminish the antimicrobial effect of nisin [10]. Hence, different strategies were developed to improve the preservative efficacy of nisin such as liposomes [11] and nanoparticles [12]. However, these reported techniques are not suitable for applications in food industries due to the utility of inorganic solvents and chemical compounds, in addition to they are expensive and complicated. For these reasons, alternative organic chemicals and solvents or green synthesized nanoparticles were developed to overcome the inactivation of free nisin by many food components through protecting nisin and releasing it in sustained manner [13]. For instance, acetic acid, a well-known biocompatible organic acid, has no adverse effects, no dietary restrictions and it is generally recognized as a safe food additive. This organic acid is commonly used, as a natural preservative, in the preservation of food especially in cheese and dairy products where it inhibit the development of bacteria, yeast and fungi [14, 15]. Besides acetic acid, tween 80 has a great potential to stabilize nanoparticles dispersion through formation of a protective coat around the nanoparticles, so it was used in food without adverse health effect [16, 17].

Application of nisin in dairy industry was reported in more than 55 countries due to its prominent antimicrobial, technological characteristics, safety, stability and flavorless. Commercially, nisin was used in several food matrices to ensure safety, extend shelf life, and to improve the microbial quality either through addition of nisin directly in its purified form or through its production in situ by live bacteria [18–20]. For instance, nisin was added as a bio-preserving ingredient in some kinds of cheese [21–23], skim milk and whole milk [24–27]. Nisin has a potent antibacterial effect against spore-forming bacteria that are the main spoilage concerns in the food industry [26]. However, several factors such as neutral pH [4], Fat% [25], protein% [28] as well as calcium and magnesium concentrations that can reduce the antimicrobial efficacy of nisin were reported when used directly in dairy foods [15, 29, 30]. Certain previously reported strategies, such as encapsulation and nano-encapsulation of nisin, were applied to increase the antimicrobial efficacy of nisin in dairy industry [31, 32]. . Importantly, there is no available data about the use of nisin or nisin NPs as antimicrobial agents during yoghurt preparation.

Accordingly, the current study was designed to prepare nisin NPs by simple nanoprecipitation technique using natural, biocompatible and safe materials. Also the aims of this study were extended to investigate the antibacterial effect of obtained nanoparticles on MRSA and *E.coli* O157:H7 during manufacturing and storage of yoghurt. Additionally, the effect of the used nisin NPs on the organoleptic properties of yoghurt was addressed.

## Materials and methods

### Materials

Acetic acid (Merck Co., Germany), nisin (Sigma Aldrich from *Lactococcus lactis*, potency ≥ 900 IU/mg, purity ≥ 95%, CAS Number 1414-45-5), Brain Heart Infusion (BHI) (BBL 11,407, USA), phosphate buffer saline (PBS) (Oxoid, Basingstoke, UK) were purchased and used as received. Polyethylene glycol sorbitan monooleate (Tween 80) was purchased from Sigma Aldrich. Additionally, Mueller Hinton agar (M173) was purchased from HiMedia (Pvt., India), and LAB204 Neogen Company. While, 0.5 McFarland Standard (8.2 log_10_ CFU/ml) (Cat. No. TM50) was purchased from Dalynn Biologicals Co. The deionized water was obtained from the Molecular Biology Unit, Assiut University, Egypt.

### Preparation of nisin nanoparticles

Nisin (2 mg/mL) was completely dissolved in 100 mL of 0.1 M aqueous acetic acid solution with the aid of sonication using cold probe sonication (UP100H Hielscher Ultrasound). Then, 50 mL of deionized distilled water was gradually added to the nisin solution while maintaining the pH value within the range of 2.5 to 3. Further, 0.01% tween 80 was added as a stabilizer and the mixture was constantly stirred at 25 oC for 7 h to eliminate acetic acid as much as possible. Finally, the nanoparticles suspensions were then sonicated for 5 min before stored at refrigerator temperature for further use. The obtained nanoparticles were examined for size, shape, antibacterial activity and stability after six months.

### Characterization of the prepared nisin NPs

#### Dynamic light scattering (DLS)

The prepared nanoparticles was characterized by DLS at a fixed scattered angle of 90° using a Zetasizer, ZS 90 (3000 HS, Malvern Instruments, Malvern, UK) at the Nanotechnology Unit, Al-Azhar University at Assiut, Egypt. Measurements were taken at 25 °C and Zetasizer® software (version 7.03) was used to collect and analyze the data [33].

#### Fourier-transform infrared spectroscopy (FTIR)

FTIR was performed at the Chemistry Department at the Faculty of Science, Assiut University. This experiment was used to identify the functional groups and the fingerprint of the molecule. Samples were prepared by compressing potassium bromide with either free nisin or NNPs into small discs. The produced discs were then scanned using FTIR spectrometer (FTIR, NICOLET, iS10, Thermo Scientific) in the wave number ranged from of 4000 to 500 cm^− 1^ [34].

#### High resolution transmission electron microscopy (HRTEM)

The morphology of the prepared nisin NPs was determined using HRTEM (JEM2100, Jeol, Japan) at the Electronic Microscope Unit, National Research Center, Egypt. The sample was diluted with deionized water, and a small drop of nisin NPs was dropped onto 200-mesh copper coated grids at room temperature and negatively stained with uranyl acetate for 3 min. Excess liquid was removed using Whatman filter paper and samples were dried at room temperature [35].

### Bacterial strains and inoculum preparation

The tested pathogens (MRSA and *E. coli* O157:H7) were previously isolated from dairy products (milk, cheese and yoghurt) samples by culture method and identified using conventional biochemical method and PCR at a certified food lab, Animal Health Research Institute (AHRI), Egypt [36, 37]. These isolates were inoculated in trypticase soy broth (Himedia, India) and incubated at 37˚C for 24 h, then co-cultured on selective agars such as MRSA agar base (Acumedia, 7420, USA) and Sorbitol MaCconkey agar (Himedia, India) [38, 39] for MRSA and *E. coli* O157:H7, respectively. The isolates were inoculated in BHI broth and incubated at 37 °C for 24 h until turbidity was comparable to a 0.5 McFarland turbidity standard. Before inoculating bacteria in milk, the inoculum was washed twice in PBS and then re-suspended in skim milk.

### Determination of minimum inhibitory concentration (MIC) of free nisin and nisin nanoparticles against MRSA and *E. Coli* O157:H7

To determine the MIC of nisin NPs against MRSA and *E.coli* O157:H7, the agar well diffusion method was used according to Suresh et al. [40] with minor modifications. In brief, 0.1 mL of the previously prepared bacterial suspensions was spread on Mueller Hinton agar plates and left for 10 min to be absorbed. Then, 8 mm wells were punched into the agar plates for testing the antimicrobial activity of nanoparticles. One-hundred µl of different concentrations of free nisin and nisin NPs (from 0.0313 mg/mL to 2 mg/mL) were poured onto the wells. One well in each plate contained 100 µL of sterile deionized water was kept as a negative control. After overnight incubation at 35 ± 2 °C, the diameters of the inhibition zones were observed and measured in mm [41]. Each concentration was performed in triplicate.

### Assessment of nisin nanoparticles cytotoxicity

The biocompatibility and the cytotoxicity of the nisin NPs were evaluated using a MTT assay against a Vero cell line after culture at 37 °C in a humidified incubator with 5% CO_2_ in Dulbecco’s Modified Eagle’s Medium supplemented with 10% Fetal Bovine Serum. The cells were seeded into a 96-well plate at a density of 1 × 10^4^ cells/well overnight before treatment. Different dilutions (0.5×MIC, MIC, 2×MIC, 4×MIC) of optimized nisin NPs were added to the seeded cells. Cells without nanoparticles served as control group. After 72 h, the consumed media was replaced with phosphate buffered saline, 10 µL from 12 mM MTT stock solution was added to each well and cells were incubated for 4 h at 37 °C. Next, 50 µL DMSO was added to dissolve formazan crystals and then the absorbance was measured at 570 nm using a BMG LABTECH®-FLUO star Omega microplate reader (Ortenberg, Germany). All experiments were performed in triplicate.

### Antibacterial efficacy of the free nisin and nisin NPs against MRSA and *E. Coli* O157:H7 during manufacturing and storage of yoghurt

Fresh milk was heated at 85 °C for 5 min in water bath then suddenly cooled. The prepared inoculums were added to the warmed milk (41 ºC) in a count of 10^6^ CFU/mL. The inoculated milk was divided into four parts for further use as following, part 1 is the positive control (contained MRSA or *E. coli O157:H7* only, one jar each), part 2 (contained MRSA or *E. coli* O157:H7 with nisin NPs at MIC and 2×MIC, two jars each), part 3 (contained MRSA or *E. coli* O157:H7 with free nisin at MIC and 2×MIC, two jars each) and part 4 (negative control; free from pathogens and contained free nisin or nisin NPs only, one jar each). After inoculation of the different treatments, yoghurt was manufactured according to Sarkar [42] by adding 2% yoghurt starter culture (*Streptococcus thermophilus* and *Lactobacillus delbrueckii* subsp. *bulgaricus*) at 41 °C to milk. The prepared yoghurt was placed in a constant-temperature incubator at 40 °C until pH reached 4.6 to 4.5. Finally, the obtained products were stored at refrigeration temperature (4 ± 1 °C) for 5 days. Samples were collected just after manufacturing of yoghurt and every 2 days during storage, then tested for the count of MRSA using MRSA agar base media [43], and *E. coli* O157:H7 using Sorbitol MacConkey (SMAC) agar plates [44]. In addition, pH values were determined in the examined samples as previously described by Igbabul et al. [45]. In brief, 10 g o f yoghurt sample was dissolved in 100 mL of distilled water. The mixture was left to equilibrate at room temperature. Then, the pH of the samples was then measured by a pH meter (Microprocessor pH meter, pH 537, WTW, Germany).

### Organoleptic assay of manufactured yogurt

Pathogen-free yoghurt jars (negative control) were prepared with two concentrations of either free nisin or nisin NPs (MIC and 2×MIC) as previously mentioned to be used for organoleptic evaluation. Thirty-five panelists were selected in teams of different ages, sex and education. The perception of consumers toward samples with two concentrations of nisin NPs was recorded. Consumers were asked to evaluate the color, flavor, mouth feel, appearance, and overall acceptability (OAA) of the prepared yoghurt samples containing nisin NPs [46]. The scale points were excellent (5); very good (4); good (3); acceptable (2); and poor (1).

### Statistical analysis

One-way analysis of variance (ANOVA) was performed using the SPSS program (SPSS Inc., Chicago, IL, USA, 18) to determine the statistical significance of differences between groups. Results with *P* < 0.05 were considered statistically significant. The microbiological and cytotoxicity assay data were prepared using Excel software version 2017. While, the FTIR results were performed using Origin Lab 2021 for graphing and analysis. All experiments were carried out in triplicate.

## Results

### Characterization of the prepared nanoparticles

The freshly prepared nisin NPs had 26.55 nm size and PDI 0.227 as determined by zetasizer. While, the diameter of the same after 6 months at refrigeration temperature was 86.50 nm with a PDI equal to 0.431 (Table 1). These results indicated that reasonable small-sized particles of nisin were obtained by precipitation technique using acetic acid. The small size of the prepared particles and the small PDI range (from 0.2 to 0.4) indicated a mono size dispersion and a good stability of the prepared nisin NPs.

**Table 1:** Physical properties of the prepared nisin NPs using Zeta-sizer

Type of nano-nisin	PDI	Size ± SD	Intensity %
Freshly prepared	0.227	26.55 ± 3.37	100%
After 6th months	0.431	86.50 ± 13.10	100%

**Fig. 1:**
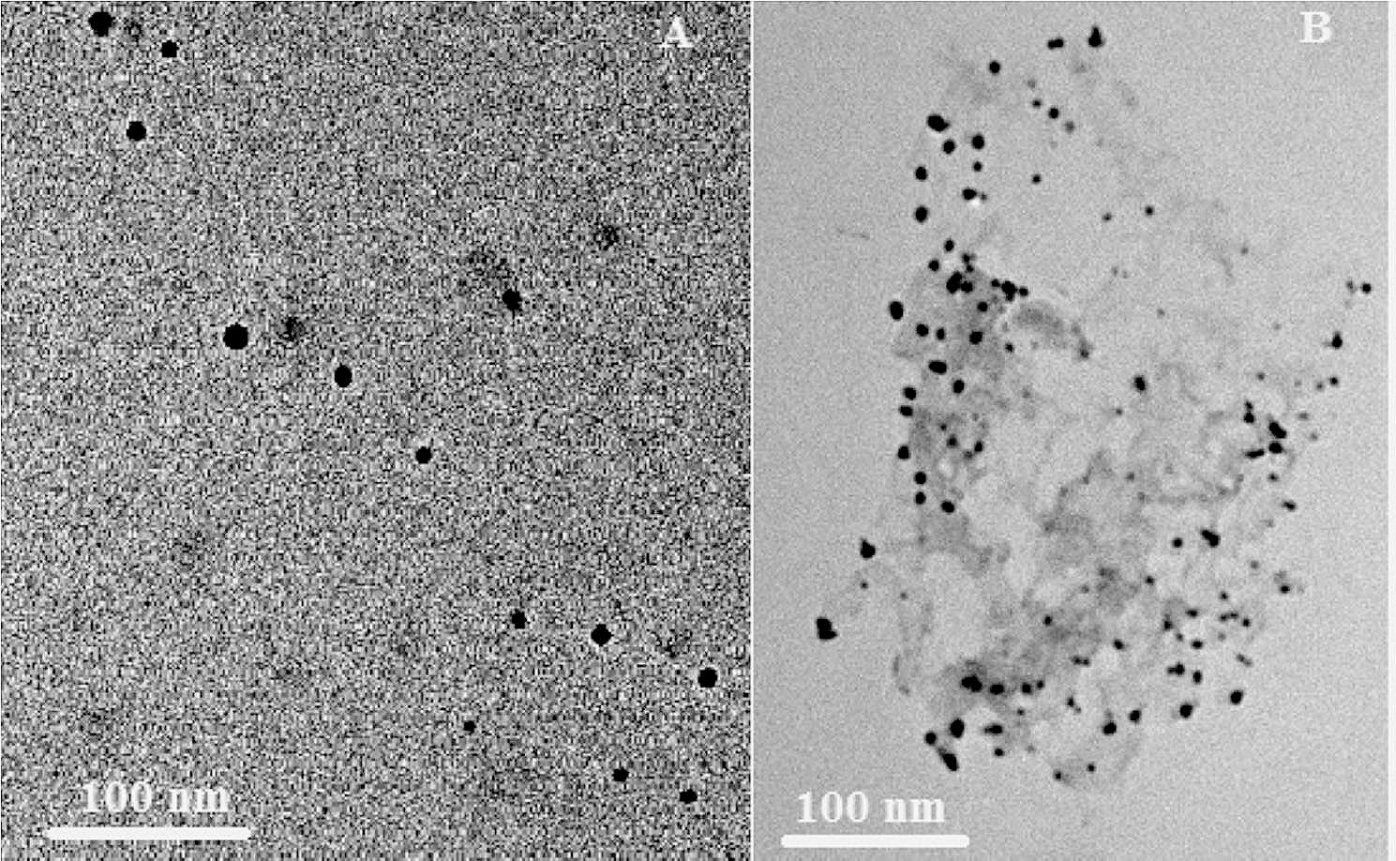
The TEM images of freshly prepared nisin NPs **(A)** and after 6th months of storage **(B)**

The size and morphology of the freshly prepared nisin NPs and after 6 months of storage were measured by HRTEM are presented in Fig. 1. Both freshly prepared and stored nisin NPs were approximately uniform in size with adequate distribution of particles. The shape of the particles was nearly spherical with slightly a bit of agglomeration just after 6 months of storage. The average size of freshly prepared nisin NPs was 7.35 nm while, after 6 months was 15.4 nm. The size of particles determined by TEM is usually smaller than the dynamic particles determined by zeta-sizer because TEM determine the actual particle diameter while zeta-sizer determine the particles diameter with adjacent moving layers of solvents.

**Fig. 2:**
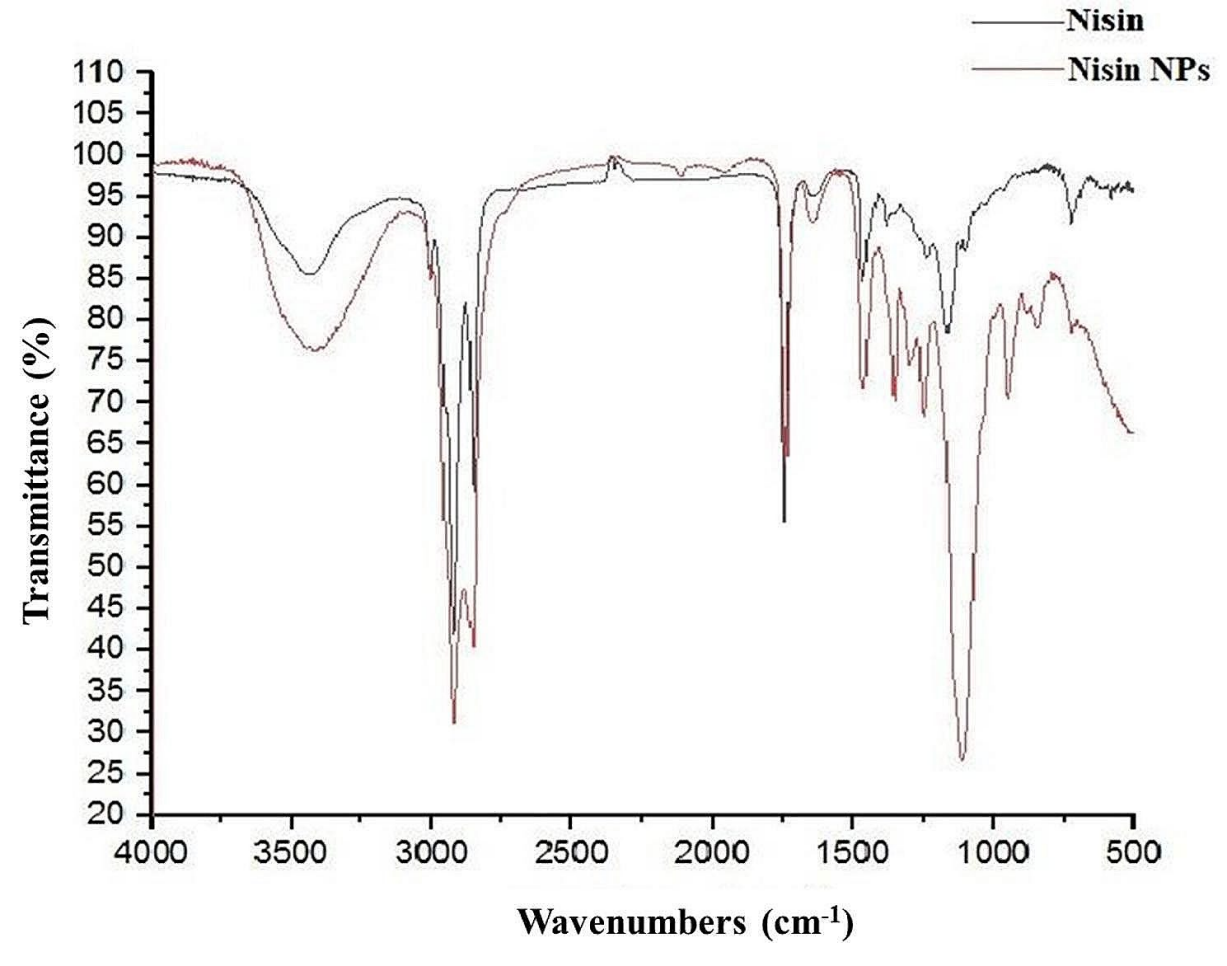
The FTIR of pure nisin and nisin NPs

Figure 2 showed the FTIR of pure and nisin NPs; both spectrum showed the characteristic peaks of nisin at 3425, 1599 and 1493 cm^− 1^ corresponded to O-H stretching of COOH, C = O stretching of amide I and N-H bending amide II. Bands 1530 cm^− 1^ in free nisin indicated the stretching of amid II and which, increased to 1549 cm^− 1^ in nisin NPs that indicated increase the H- bond in nano form than free one. The results of FTIR spectrum confirmed that the formation of nisin NPs did not result in any chemical changes or interaction of nisin with used the materials. These results also demonstrated the suitability of the applied method for the preparation of chemically stable and small-sized nisin NPs.

### Assessment of Nisin nanoparticles cytotoxicity

**Fig. 3:**
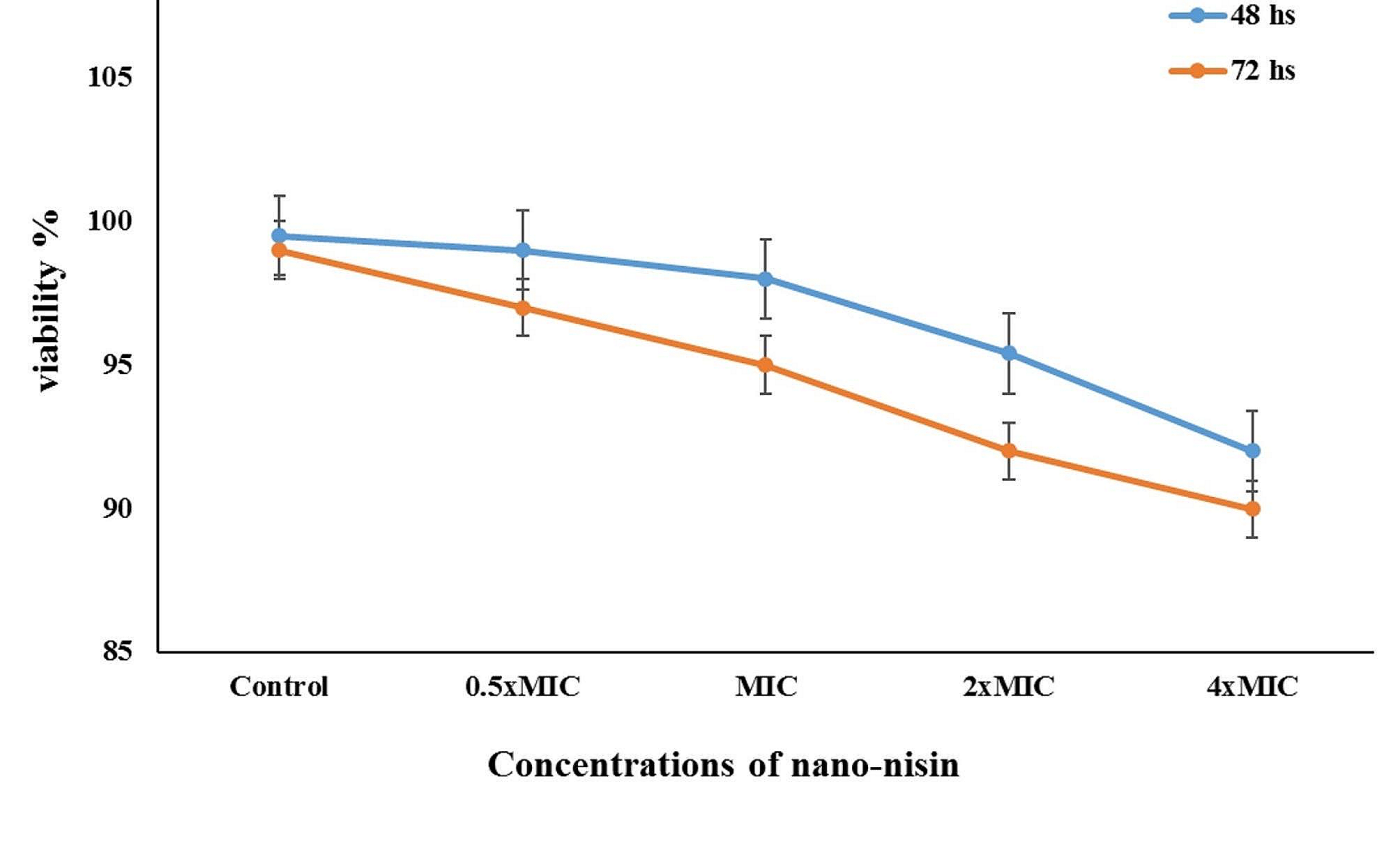
Cytotoxicity and cell viability of different concentrations nisin NPs using Vero cells after 48 and 72 h using MTT assay

In the present study, Veros cells were exposed to nisin NPs for 48 and 72 h, and the cytotoxicity was measured by MTT assays. Results showed that the MIC did not exhibit an anti-proliferation effect (Fig. 3). Interestingly, even at very high concentrations (4xMIC), there were no cytotoxicity effect as the percentage of viable cells reach 92% and 89.98% after 48 and 72 h, respectively. The obtained findings confirmed the safety and good biocompatibility of the prepared nisin NPs at MIC level.

### MIC of free nisin and nisin NPs against MRSA and *E. Coli* O157:H7

The efficacy of the free nisin and prepared nisin NPs against MRSA and *E. coli* O157:H7 was investigated using agar well diffusion assay (Table 2). Nisin and its nanoparticles showed potent antibacterial effect against MRSA than *E. coli* O157:H7. The MICs of nisin and nisin NPs toward MRSA were 0.0625 and 0.0313 mg/mL, respectively. While, 0.125 mg/mL was the MIC of both nisin and nisin NPs against *E. coli* O157:H7. Of note, growth inhibition zone was not observed against MRSA at 0.0313 mg/mL of nisin, and toward *E. coli* O157:H7 at both 0.0625 and 0.0313 mg/mL nisin (Table 2). On the other hand, the prepared nisin NPs could produce inhibition zones against MRSA with a mean diameter ranged from 25.4 ± 2.1 mm to 7.1 ± 0.89 mm at concentrations of 2 to 0.0313 mg/mL, respectively. Also, the nisin NPs showed anti-*E. coli* O157:H7 activity at different concentrations of 2, 1, 0.5, 0.25 and 0.125 mg/mL with average size of 20.1, 15.4, 12.7, 9.5 and 7.2 mm of the inhibitory zones, respectively. There were no inhibition zones against *E. coli* O157:H7 at 0.0625 and 0.0313 mg/mL of nisin NPs. Overall, the obtained findings indicated that the most effective MICs of nisin and nisin NPs for both organisms were 0.125 mg/mL (Table 2).

**Table 2:** Antimicrobial activity (MIC) of the evaluated materials (pure nisin and nisin NPs) against foodborne pathogens (MRSA and *E. coli* O157:H7), as detected in the well diffusion assay (mm)

Antimicrobial substances	Concentrations (mg/mL)	The diameter (mm)	
		MRSA	*E. coli* O157:H7
**Pure nisin**	2	20.4 ± 3.5^a^	19.0 ± 3.0^a^
	1	19 ± 1.9^a^	13.3 ± 2.9^a^
	0.5	17 ± 1.5^a^	10.5 ± 2.5^ab^
	0.25	15.2 ± 1.2^a^	8.0 ± 3.0^b^
	0.125	11.3 ± 1.1^ab^	6.7 ± 2.0^b^
	0.0625	7.8 ± 1.0^b^	0.0 ± 0.0^c^
	0.0313	0.0 ± 0.0^c^	0 ± 0.0^c^
**Nisin NPs**	2	25.4 ± 3.7^a^	20.1 ± 2.5^a^
	1	22.8 ± 3.0^a^	15.4 ± 2.0^a^
	0.5	19.5 ± 2.7^a^	12.7 ± 1.5^a^
	0.25	16.45 ± 2.0^ab^	9.5 ± 2.0^b^
	0.125	11.32 ± 1.5^b^	7.2 ± 1.0^b^
	0.0625	8.64 ± 1.5^b^	0.0 ± 0.0^c^
	0.0313	7.1 ± 1.0^bc^	0.0 ± 0.0^c^

Inhibition zones expressed as the mean of three replicates ± SD

### Antibacterial effect of nisin and nisin NPs against MRSA and *E. Coli* O157:H7 during manufacturing and storage of yoghurt

**Fig. 4:**
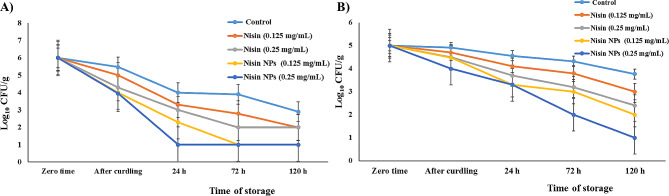
Antibacterial effect of free nisin **(A)** and nisin NPs **(B)** on MRSA and *E.coli* O157:H7 during manufacturing and storage of yoghurt

Figure 4 presented the antibacterial activity of nisin against the examined foodborne pathogens (MRSA and *E. coli* O157:H7). Here, nisin at 0.125 and 0.25 mg/ml could induce antibacterial effect against MRSA (3.3 and 3 log_10_ CFU/g, respectively) after 24 h of yoghurt storage. However the effect was not higher as in case of nisin NPs (2.3 and 1 log_10_ CFU/g) at the same concentrations and time of storage. While, the inhibitory impact of the free nisin on *E. coli* O157:H7 was observed after 24 h (3.7 log_10_ CFU/g) and 3 days (3.8 log_10_ CFU/g) of storage at the concentrations of 0.25 and 0.125 mg/mL, respectively. The pathogens were still detected till the end of the experiment in nisin treated yoghurt (Fig. 4).

**Fig. 5:**
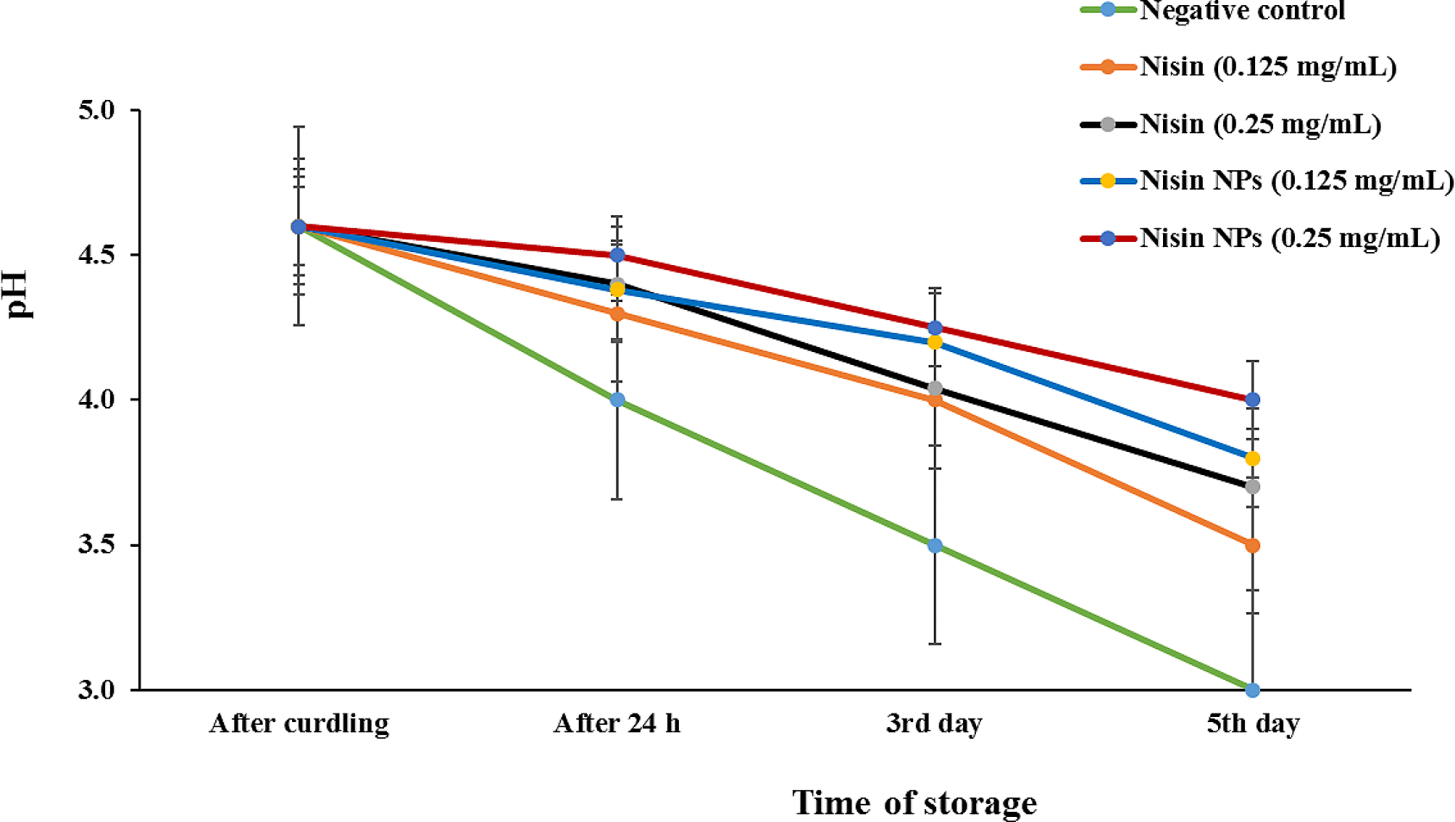
Evaluation of pH levels during processing and storage of yoghurt inoculated with different concentrations of free nisin or nisin NPs

On the other hand, there was a clear reduction in mean count of MRSA and *E.coli* O157:H7 in the laboratory-manufactured yoghurt supplemented with different concentrations (0.125 and 0.25 mg/mL) of nisin NPs. A complete inhibition of MRSA was observed after 24 h and at the 3rd day of storage by 0.25 and 0.125 mg/mL of nisin NPs, respectively (Fig. 5). While, *E. coli* O157:H7 was undetectable at the 5th day of storage with 0.25 mg/mL nisin NPs, however it was still detected till the end of the experiment in either yoghurt inoculated with 0.125 mg/mL nisin NPs or in the positive control group (Fig. 4). Taken together, the antimicrobial count tests revealed that the free nisin is not effective as the nisin NPs at same time points during processing and storage of yoghurt.

During storage, the pH did not change significantly between different treatments. However, the negative control group showed little decrease in pH in comparison to other groups at the 3rd and 5th day of storage (3.5 and 3, respectively).

### Organoleptic evaluation of the laboratory-manufactured yoghurt

**Fig. 6:**
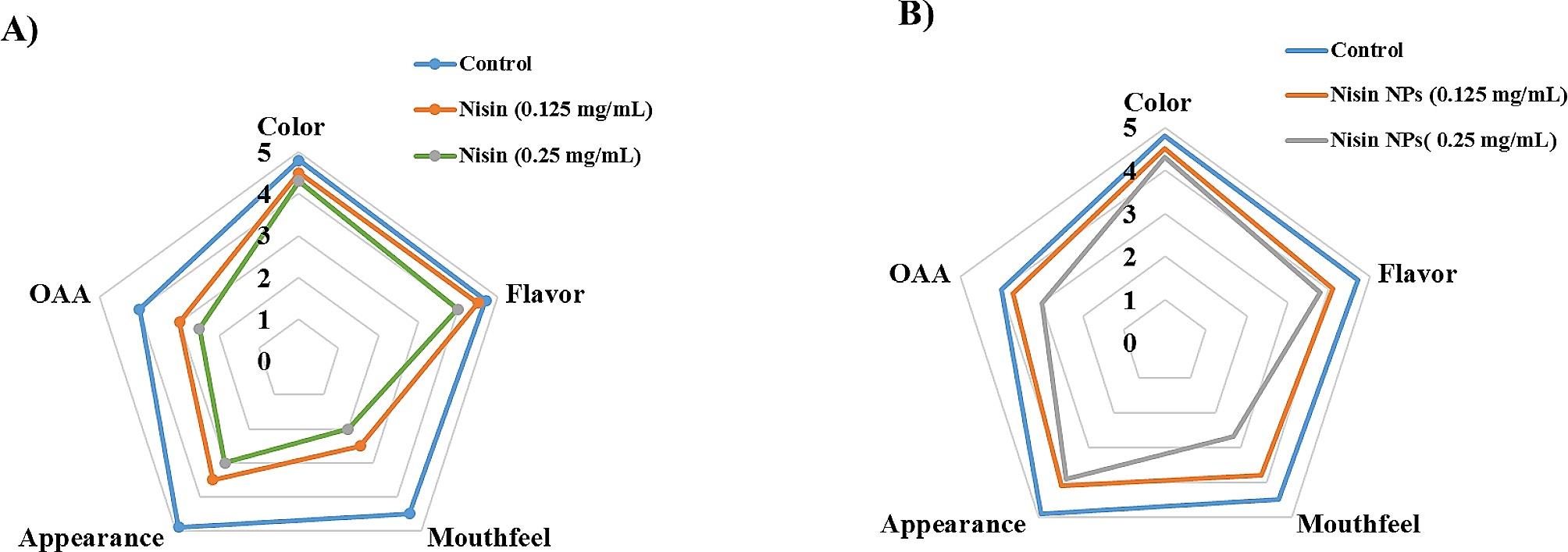
Organoleptic properties of yoghurt inoculated with different concentrations of free nisin and nisin NPs

Figure 6 clarified that there was no difference in the sensory properties between the different groups (contained 0.125 or 0.25 mg/mL nisin (Fig. 6A) or nisin NPs (Fig. 6B)) in comparison to the control group. The OAA of yoghurt inoculated with 0.125 mg/mL and 0.25 mg/mL of free nisin was 3 and 2.5, respectively (Fig. 6A). While, the control samples had the highest score in mouth feel (4.5), followed in order with yoghurt loaded with 0.125 mg/mL and 0.25 mg/mL nisin NPs (3.8 and 2.7, respectively). Additionally, the overall acceptability (OOA) of control, 0.125 mg/mL and 0.25 mg/mL nisin NPs groups was 4, 3.7 and 3, respectively (Fig. 6B). Such findings indicated the high acceptability of yoghurt containing different concentrations of nisin NPs than those inoculated with free nisin.

## Discussion

The current study elucidated for the first time the inhibitory effect of free nisin and nisin NPs on two of the most common foodborne pathogens (MRSA and *E. coli* O157:H7) during processing and storage of laboratory manufactured yoghurt. Strikingly, adding of nisin NPs to yoghurt could induce much higher antibacterial effect on MRSA and *E. coli* O157:H7 with high consumer acceptability than free nisin. Accordingly, nisin NPs could be a useful and effective bio-preservative candidate against MRSA and *E. coli* O157:H7 in dairy industry.

The present study revealed that nisin NPs was prepared by a novel and safe method using natural material such as acetic acid which is commonly applied in food products. Chang et al. [47]. prepared ultra-small sizes of nisin NPs by nanoprecipitation method using HCL while we obtained much smaller particle size of NNPs using acetic acid which is more safer, less toxic and accepted by consumers. The particle size determined by TEM is smaller than the size measured by DLS this difference could be attributed to the removal of solvent and shrinking of nanoparticles during the drying of nisin NPs samples for TEM investigations. In addition, DLS measures the hydrodynamic diameter of the dispersed moving particles with the surrounding moving layers of solvents [48, 49].

The result of FTIR was in consistent with that of Flynn et al. [50]. Herein, we found that the -OH stretching peak of nisin NPs displayed a greater intensity than that of free nisin, which indicated a stronger hydrogen bonding formation within nisin NPs. In case of free nisin, the peak at 1620 cm^− 1^ corresponding to COO^−^ was shifted to 1610 cm^− 1^ in nisin NPs indicating that the hydrogen bonding was increased within nisin NPs. In contrast, the amid II band in free nisin appeared at 1530 cm^− 1^ became more obvious at 1549 cm^− 1^ in nisin NPs which was in agreement with Webber et al. [51]. . Band of amide I at wave number of 1632 cm^− 1^could be due to the change in the structure of free nisin when converted into nisin NPs by using natural acetic acid.

In food chain, nisin has been approved for use in over 50 countries due to its safety and its potent antimicrobial activity without inducing microbial resistance [52]. Of particular note, the FAO/WHO Codex Committee and US FDA allow using nisin as a food additive in dairy products at a concentration up to 250 mg/kg [1, 53]. Moreover, European Food Safety Authority [54] reported that nisin has been shown to be non-toxic to humans and it is safe as a food preservative for dairy and meat products. In the current study, the examined organisms (MRSA and *E. coli* O157:H7) have been involved in many food outbreaks worldwide as well as their resistance to many antibiotics, considered a challenge to be controlled [55–57]. Therefore, the present study could be a useful alternative strategy to avoid the possible health hazards of these organisms after consumption of yoghurt using either nisin or nisin NPs as natural food preservatives.

The obtained results revealed that the MICs of nisin and nisin NPs against MRSA were lower than that of *E. coli* O157:H7. This could be due to the ability of nisin to penetrate the cell wall of Gram-positive bacteria, however, it is difficult for nisin to penetrate the outer membrane barrier of Gram-negative bacteria [58]. Nisin could destroy bacteria through two mechanisms, either by making pores in the plasma membrane or by inhibiting the cell wall biosynthesis through binding to lipid II [59–61]. Importantly, the obtained results in the current study showed that that MIC of nisin NPs against MRSA was lower than that of pure nisin. Similarly, Zohri et al. [62] reported that the MICs of nisin and Nisin-Loaded nanoparticles was 2 and 0.5 mg/mL after 72 h of incubation period with the *S. aureus* samples, respectively. In addition, Moshtaghi et al. [63] examined the antibacterial effect of nisin on *S. aureus* and *E. coli* at different pH values and they found that the MICs against *S. aureus* were ranged from 19 to 312 µg/mL of nisin at pH levels from 8 to 5.5, respectively. While for *E. coli*, the MICs were from 78 to 1250 µg/mL at the same range of pH, respectively [63].

Interestingly, nisin inhibited the pathogenic foodborne bacteria and many other Gram-positive food spoilage microorganisms [13]. In the present study, evaluation of the kinetic growth of MRSA and *E. coli* O157:H7 based on the total counts in the laboratory manufactured yoghurt revealed that nisin NPs was able to inhibit more effectively the growth of such foodborne pathogens than free nisin during manufacturing and storage of yoghurt. These findings were in concurrent with those obtained by Zohri et al. [62] who demonstrated that nisin-loaded chitosan/alginate nanoparticles showed more antibacterial effect than free nisin on the growth of *S. aureus* in raw and pasteurized milk samples. Additionally, nisin Z in liposomes can provide a powerful tool to improve nisin stability and inhibitory action against *Listeria innocua* in the cheddar cheese [64]. In our study, nisin NPs showed a complete inhibition of MRSA after curdling of yoghurt and reduced the survivability of *E. coli* O157:H7 when applied at two different concentrations during storage of such product. Nisin NPs with high specific surface area could be easily attached to the target cell surface leading to increased permeability of the cell membrane, and finally cause bacterial cell death. Furthermore, nisin NPs were thermo-tolerant because of the internal non-covalent interactions in the nanoparticles [4, 65]. Additionally, the decline in the mean count of the examined pathogens (MRSA and *E.coli* O157: H7) in the current study may be due to the effect of low pH (high acidity) of yoghurt that leads to shrinkage and death of the bacterial cells [66]. Similarly, Al-Nabulsi et al. [67] reported that the combination of a starter culture, low temperature, and pH (∼5.2) had inhibitory effects on the growth of *S. aureus*.

The effect of adding different levels of nisin and nisin NPs on OAA scores of yoghurt was recorded and the obtained results were in agreement with Hussain et al. [68], Radha [3], and Gharsallaoui et al. [4] who reported that a Nigerian fermented milk product had acceptable sensory scores till 25th day of storage when loaded with nisin at 400 IU/mL. Additionally, Chang et al. [47] said that the thermal treatments are known to cause undesirable changes in the sensory, nutritional and/or technological properties of milk. Taking advantage of the antimicrobial action of nisin NPs against several spoilage and pathogenic microorganisms, this innovative non-thermal food preservative offers the inactivation of microorganisms with minimal impact on the quality, safety, nutritional values and acceptability of dairy products.

Overall, as the demand for preservative-free food products increased, natural antimicrobials have gained more and more attention because of their effectiveness and safety. Consequently, the current study investigated that the addition of nisin NPs to milk for manufacturing of yoghurt can be used as an innovative preventive measure to inhibit the contamination with foodborne pathogens. However, further researches are required to determine the effective and safe dose of nisin NPs for application in other dairy products.

## Conclusion

The present study prepared nisin NPs using acetic acid by precipitation method and the obtained particles were small in size with good stability and consumer acceptability. The antibacterial effect of nisin and nisin NPs against MRSA and *E. coli* O157:H7 in yoghurt was impressive. Additionally, the studied nanoparticles did not affect the sensory and textural characteristics of the finished product. Hence, this study could be useful for yoghurt makers and dairy products factories through using this novel preservation technology to inhibit the growth of MRSA and *E. coli* O157:H7, in yoghurt and dairy products, and subsequently avoid food spoilage and foodborne diseases.

## Data Availability

All data and materials are available here in the current study.
